# Hair Measurements of Cortisol, DHEA, and DHEA to Cortisol Ratio as Biomarkers of Chronic Stress among People Living with HIV in China: Known-Group Validation

**DOI:** 10.1371/journal.pone.0169827

**Published:** 2017-01-17

**Authors:** Shan Qiao, Xiaoming Li, Samuele Zilioli, Zheng Chen, Huihua Deng, Juxian Pan, Weigui Guo

**Affiliations:** 1 Department of Health Promotion Education and Behavior, School of Public Health, University of South Carolina, Columbia, South Carolina, United States of America; 2 Department of Psychology, Wayne State University, Detroit, Michigan, United States of America; 3 Research Center for Learning Science, Southeast University, Nanjing, China; 4 Beihai Center of Disease Control and Prevention, Beihai, Guangxi, China; University of Marburg, GERMANY

## Abstract

**Background:**

Existing literature suggests that endocrine measures, including the steroid hormones of cortisol and Dehydroepiandrosterone (DHEA), as well as the DHEA to cortisol ratio in the human hair can be used as promising biomarkers of chronic stress among humans. However, data are limited regarding the validity of these measures as biomarkers of chronic stress among people living with HIV (PLWH), whose endocrine system or hypothalamic pituitary adrenal (HPA) axis may be affected by HIV infection and/or antiretroviral therapy (ART) medications.

**Method:**

Using hair sample data and self-reported survey from 60 PLWH in China, we examined the validity of three endocrine measures among Chinese PLWH using a known-groups validation strategy. High-stress group (n = 30) and low-stress group (n = 30) of PLWH were recruited through individual assessment interviews by a local licensed psychologist. The endocrine measures in hair were extracted and assessed by LC-APCI-MS/MS method. Both bivariate and multivariate analyses were conducted to examine the associations between the endocrine measures and the stress level, and to investigate if the associations differ by ART status.

**Results:**

The levels of endocrine measures among Chinese PLWH were consistent with existing studies among PLWH. Generally, this pilot study confirmed the association between endocrine measures and chronic stress. The high stress group showed higher level hair cortisol and lower DHEA to cortisol ratio. The higher stress group also reported higher scores of stressful life events, perceived stress, anxiety and depression. Hair cortisol level was positively related to anxiety; DHEA was negatively associated with stressful life events; and the DHEA to cortisol ratio was positively related to stressful life events and perceived stress. ART did not affect the associations between the endocrine measures and stress level.

**Conclusions:**

Our findings suggest that hair cortisol and DHEA to cortisol ratio can be used as promising biomarkers of chronic stress among PLWH. Clarifying the role of steroid hormones in the psychoimmunology of PLWH may yield important implications for clinical practice and psychological intervention.

## Introduction

HIV (Human Immunodeficiency Virus), the virus that causes AIDS (Acquired Immunodeficiency Syndrome), has become one of the most serious health and developmental challenges for humans since it was reported in 1981 [1]. Worldwide there were 35 million people living with HIV (PLWH) and 1.5 million deaths were attributed to the condition in 2013 [2]. In addition to physical health complications, people affected by HIV are often stigmatized and face recurrent discrimination across a variety of social realms, including work and health care settings [3]. This combination of chronic psychological and physical stressors can have daunting consequences for individual psychological wellbeing, which in turn can further exacerbate physical symptoms [4]. The risk of having mental health problems is higher in PLWH than general population. Globally, an estimated 350 million people of all ages suffer depression by 2015, accounting for 5% of the total population in the world [5]. One recent literature review suggested that the mean prevalence of depression and anxiety among HIV patients was 33.6% and 28.38% respectively, with a higher prevalence in low and middle income counties (41.36% and 33.92%, respectively)[6]. Psychological problems may have adverse effects upon medication adherence and other HIV-related outcomes [7]. Within this context, it becomes a priority to develop interventions aimed at helping PLWH mitigate stress. However, a fundamental requirement for developing effective interventions is to identify reliable indicators that provide a comprehensive psychobiological profile of an individual’s stress level. This can be achieved through the use of multifaceted measures of stress, ranging from self-report assessments to physiological indicators. While the self-report data are valuable, objective and reliable biological indicators (biomarkers) will provide stronger evidence in both exploratory studies and evaluation of interventions. The examination of the association between chronic stress and hair endocrine level (cortisol, DHEA, and DHEA to cortisol ratio) will contribute to identifying and validating novel biomarkers for future studies.

The hypothalamic-pituitary adrenal (HPA) axis serves as the body’s central stress response system. It is a complex set of direct influences and feedback interactions among three endocrine glands: the hypothalamus, the pituitary gland, and the adrenal glands [8]. Its end product, cortisol, has been widely used as one of the main indicators of an individual’s cumulative physiological risk associated with psychological stress [9, 10]. Cortisol plays a critical role in the coordination of brain and body functioning when confronted with a stressor [11]. Cortisol levels rise at times of stress, allowing the body to mobilize resources rapidly during activation of the sympathetic division of the nervous system; levels also serve to assist in counterbalancing the body’s response in the long term [12].

The most common sampling matrices from which cortisol concentrations can be obtained are blood (serum or plasma) and saliva. Because these approaches provide measures of cortisol concentration at single points in time, they are significantly affected by the time of the day samples are collected, individual differences in circadian rhythms, and transient exposure to acute daily stressors [13]. Multi-time-point, multi-day sampling of serum or saliva might help overcome these limitations, but these sampling procedures can be very expensive and face many logistical challenges (e.g., participant compliance)[14].

Recently, promising findings have emerged that suggest the feasibility of extracting cortisol concentrations from hair samples [15], akin to the established practice of extracting drug concentrations from hair strands [16]. Deposition of cortisol in the hair follicles is constant. For this reason, hair cortisol concentrations provide a measurement of long-term HPA activity, thus serving as an indicator of the systemic HPA activity over the long-term (i.e., weeks and months), and therefore hair cortisol is a potentially promising biologic marker of chronic stress [17].

Another steroid hormone worth investigating when assessing levels of chronic stress is Dehydroepiandrosterone (DHEA). DHEA is also secreted by the adrenal glands but, contrary to cortisol, tends to decline with aging [18, 19]. DHEA is also strongly implicated in the stress response system because of its stimulatory effects on catecholamine synthesis and secretion as well as its anti-inflammatory and anti-glucocorticoid effects that occur both at the molecular and physiological level [20]. Thus, DHEA plays a pivotal role in modulating physiological regulatory systems involved in the stress response, in particular the HPA axis, and several studies have found DHEA levels to be negatively correlated with chronic stress [21–23]. Because of the opposite effects of DHEA and cortisol, a common measure employed to test the impact of both hormones simultaneously is the ratio between DHEA and cortisol [24, 25]. This measure is considered an index of anabolic/catabolic balance, with lower levels (or decreases over time) associated with higher susceptibility to dysfunction of the HPA axis.

This framework thus suggests that endocrine measures including cortisol, DHEA, and the DHEA to cortisol ratio in the human hair may be promising candidates for obtaining objective measures of chronic stress in PLWH. However, within this sample, a specific challenge is to evaluate these endocrine stress profiles while considering the impact of medical treatments PLWH have to undergo. Existing literature suggests that multiple medications used to treat HIV affect adrenocortical function. Both Ketoconazole, by inhibiting cortisol biosynthesis, and rifampin, by increasing the metabolic clearance of cortisol, may lead to adrenal insufficiency in patients with impaired adrenal reserve. Megestrol acetate has been shown to suppress the HPA axis [26]. One study reported that although PLWH had higher serum levels of cortisol and lower levels of DHEA than regular healthy controls, within group variability in these hormones was not affected by antiretroviral therapy (ART) [27]. More empirical studies are needed to further explore the role of ART.

A recent study conducted in Netherland suggested a higher level of hair cortisol in PLWH compared to healthy controls [28]. However, to the best of our knowledge, no empirical studies have yet explored the link between chronic stress level and the three endocrine measures in hair among PLWH. In addition, it is not clear whether receiving ART may affect endocrine levels in hair. Therefore, using a known-groups validation methodology [29], the current study aims to examine if endocrine measures in hair can be a valid biomarker for chronic stress among PLWH in China with focuses on the following three research questions: 1) Do the endocrine measures in hair differ between PLWH with different levels of stress? 2) Are the endocrine measures in hair associated with self-reported psychological measures of stress and other relevant psychological variables (e.g., depression)? 3) Does ART affect the endocrine measures in hair (e.g., whether the associations between the endocrine measures and the stress level differ by ART status)?

## Methods

### Subjects

The study protocol was approved by the Institutional Review Board at Beihai Center for Disease Control and Prevention (CDC) in China. The written informed consents also have been obtained from the participants. The participants of this study were recruited from PLWH in Beihai city in Guangxi China. HIV patients at least 18 years of age were eligible for participation. Individuals with any of the following characteristics were excluded: 1) with known symptoms of opportunistic infections; 2) having endocrine disease (e.g., adrenal dysfunction, autoimmune thyroid diseases, hyperamylasemia, macroamylasemia, etc.); 3) taking hormonal drugs with probable influences on cortisol or DHEA levels; and 4) known history of drug use.

Research staff including HIV case managers from the Beihai CDC approached potential participants from a pool of known PLWH in the city. To construct the known stress groups (e.g., two groups with different levels of possible chronic stress), a licensed psychologist conducted a 30-minute screening and in-depth interview with each of the potential participants to determine their eligibility and also assessed their level of stress based on recent physical health condition, socio-economic status, recent stressful life events, and general psychosocial condition. Finally, we recruited 30 PLWH from those who were considered as having high psychological stress (“high-stress group”) and 30 PLWH from those who were considered as having low psychological stress (“low-stress group”) based on the results of individual psychological assessment. We also tried to match the two groups to a maximal extent in terms of age, gender, and ART uptake.

### Data collection procedure

Hair samples were collected from each participant by research staff from the Beihai CDC. Following a standardized protocol, a 1-cm hair sample (20–30 strands of hair) was cut as close to the scalp as possible from the vertex posterior region. The hair strands were cut with iron scissors that had been wiped with an alcohol pad. The hair thatch then was completely enclosed by a piece of foil and a small label indicating the study ID number was placed over the distal end of the hair thatch.

After the hair sample collection, all the participants completed an assessment inventory including basic demographic and socio-economic information, and psychosocial outcomes (e.g., stressful life events, anxiety, perceived stress, and depression). The survey lasted about 30 minutes and was carried out through a one-on-one interview. Interviewers were health care workers from the Beihai CDC who received intensive training on research ethics and individual interviews with PLWH prior to the data collection.

### Cortisol and DHEA assays

Hair samples were washed twice with 1 mL methanol for 2 minutes and then dried at room temperature for at least 12 hours. A sample of 20 mg hair was then incubated in 1 mL methanol for 24 h at 25°C in the presence of 1 ng cortisol-d4 as internal standard (IS). After being centrifuged at 12,000 rpm for 5 minutes, 800 μL the clear supernatant was transferred into a new 2 mL tube and evaporated under nitrogen at 50°C. The dry residue was re-suspended by 50 μL deionized water and 950 μL methanol for solid-phase extraction. The eluate finally obtained was evaporated to dryness and re-suspended in 50 μl mobile phase for the next analysis [30].

The detection of hair cortisol and DHEA was done by liquid chromatography tandem mass spectrometry (3200 QTRAP, ABI, USA). Cortisol and DHEA were ionized with an atmospheric pressure chemical ionization source (APCI), and identified in positive ion mode using the multiple reaction monitoring mode and quantified with internal standard method. Nitrogen (99.999%) was selected as the nebulizing gas. The optimum ionization parameters followed protocol reported in previous literature [31].

The present LC-APCI-MS/MS method showed the limits of detection (LOD) and quantification (LOQ) at 0.25 and 0.50 pg/mg for hair cortisol and at 1.25 and 2.50 pg/mg for DHEA where LOD and LOQ were defined as the concentrations with a signal-to-noise ratio (S/N) of 3 and 10. Linearity was achieved at 0.5–250 pg/mg (r^2^ = 0.9983) for hair cortisol and at 2.5–250 pg/mg (r^2^ = 0.9967) for DHEA. Intra-day and inter-day precisions and recovery (n = 5) were evaluated at 0.5, 5, and 200 pg/mg. Intra-day and inter-day coefficients of variation were less than 10% at the three concentrations and recovery was more than 98%.

### Measures

#### Demographics

Participants provided information about individual and family characteristics including gender, age, ethnicity, height, weight, religion, marital status, place of original residence, education attainment, work status, size of the family, number of children, monthly household income, and HIV-related information (e.g., time since HIV diagnosis, HIV-infection status among other family members). Participants also reported their estimated weight and height. We calculated Body Mass Index (BMI) based on weight and height for each participant using an established formula [32] BMI = weight (kg)/ [height (m)]^2^.

#### Psychological measures

Stressful life events (SLE). Participants were asked the frequency of 18 stressful life events over the previous three months (i.e., never, once, and at least twice). Sample events included death in the family, relationship problems, job loss, financial hardship and physical or sexual assault. Responses were summed, and higher scores indicated greater frequency of stressful life events (Cronbach α = .69). This measure instrument was adapted based on the scale of traumatic events used in our previous studies in China [33].

Anxiety. We employed the Zung Self-rating Anxiety Scale (SAS) to measure anxiety level. The SAS is a 20-item scale that assesses symptoms of anxiety with a 4-point response option (e.g., “how often do you feel heart pounding or racing”, “how often do you fear of losing control”). SAS has strong psychometric properties (Cronbach α = .93) and has been widely used in various Chinese populations [34].

Perceived Stress. Participants’ subjective experience of chronic stress was measured by the 14-item Perceived Stress Scale (PSS)[35], which has been validated for use in a wide variety of populations [36]. The PSS is a global measure of subjective stress that assesses the extent to which respondents perceive their lives as being unpredictable, uncontrollable, or overwhelming in the last month. Each item is rated on a 5-point scale ranging from “almost never” to “almost always” with higher scores indicating higher levels of perceived stress (Cronbach α = .78).

Depression. The Center for Epidemiological Studies Depression Scale (CES-D) was employed to measure depressive symptoms during the past week [37]. The CES-D is a 20-item self-report depression measure with a 4-point response option (0 = not at all, 1 = a little, 2 = some, 3 = a lot). A sum score was used with higher scores indicating greater levels of depression (Cronbach α = .92).

### Data analysis

Descriptive statistics were employed to examine distributions of demographic, endocrine measures (i.e., hair cortisol, DHEA, and DHEA to cortisol ratio), and psychological measures of the participants. One-way ANOAVA (for continuous measures) and Chi-square tests (for categorical measures) were used to compare differences between the two known stress groups in terms of the demographic, endocrine and psychological measures.

We examined the Pearson correlations between endocrine and psychological measures. To assess the differences of psychological measures between different levels of endocrine measures, endocrine measures were categorized into to two sub-groups (high vs. low) using median-split. Specifically, we generated hair cortisol group (“low group”: ≤13.91, “high group”: >13.91), DHEA group (“low group”: ≤31.62, “high group”: >31.62), and DHEA to cortisol ratio group (“low group”: ≤2.01, “high group”: >2.01). T-tests were employed to examine the associations between psychological measures and endocrine measures.

We used GLM analysis to further explore if ART might affect the association between endocrine measures and stress level (i.e., the two known stress groups) among PLWH. In the GLM analyses, the dependent variables were endocrine measures, and the between-subjects factors included known stress group and ART status. A significant interaction term between stress group and ART status in the GLM analysis would be an indication of a potential moderation effect of ART on the associations between stress level and endocrine measures. We also conducted t-tests and correlation analysis to further identify potential covariates (e.g., gender, age, BMI, etc.) for the GLM model. Because of the relatively small sample size and exploratory nature of the study, we did not employ a prior level of statistical significance (e.g., α = .05) but reported actual p-value associated with various statistical tests to avoid any over-interpretation of the statistical results. All statistical analyses were performed using SPSS 16.0 (SPSS Inc, Chicago, IL).

## Results

### Background characteristics

One participant had a hair cortisol value of 370 pg/mg that was considerably higher than the average (15.75 pg/mg), and thus was identified as an outlier and excluded from further statistical analyses. As presented in Table 1, nearly 58% of the participants were males. The average age was 33.3 years (SD = 8.60) and the average BMI was 20.97 (SD = 2.62). The majority of the participants (90%) were of Han ethnicity. Around 70% were currently married. The mean educational attainment was 8.84 years (SD = 4.24). The majority (70%) of the participants were born in rural areas (based upon a rural household registration or Hukou) yet only a low proportion (37%) currently lived in rural areas. There were on average 2.81 members in each family (SD = 1.88) including 1.22 young children (children who are younger than 18 years old)(SD = .91). Among the participants, 37% reported that they did not work and their monthly household income was less than 1,000 yuan (approximately 160 US dollars). The average duration of their HIV diagnosis was 2.66 years (SD = 2.11). In addition, 29% of the participants reported that at least one of their family members was also infected by HIV.

**Table 1:** Demographic characteristics, endocrine and psychological measures by stress groups.

	Overall	High Stress	Low Stress	p-value
**N (%)**	59 (100%)	29(49%)	30(51%)	
**Gender (male)**	34(58%)	16 (55%)	18 (60%)	.795
**Age in years (SD) [18–49]**	33.29 (8.60)	31.59 (8.19)	34.93 (8.81)	.136
**BMI(SD)[16.33–28.13]**	20.97(2.62)	20.10(2.51)	21.82(2.48)	**.010**
**Han ethnicity**	53(90%)	26 (90%)	27 (90%)	.572
**Marital status**				
Ever married	41 (70%)	19 (68%)	22 (73%)	.770
Never married	18 (31%)	10 (35%)	8 (27%)	
**Years of formal schooling (SD)**	8.84 (4.24)	7.94 (4.63)	9.72 (3.69)	.106
**Local residents**	34 (58%)	19 (66%)	15 (50%)	.430
**Rural Hukou**	41 (70%)	20 (69%)	21 (70%)	.635
**Still living in rural area**	22 (37%)	11 (38%)	11 (37%)	.625
**Number of people in family (SD)**	2.81 (1.88)	2.66 (2.02)	2.97 (1.75)	.529
**Children**				
Have any children	32 (54%)	13 (45%)	19 (63%)	.195
No. of adult children (SD) [0–3]	.69 (1.06)	.46 (.97)	.84 (1.12)	.327
No. of young children (SD)[0–4]	1.22 (.91)	1.62 (1.04)	.95 (.71)	.038
**Work status**				
Full time	20 (34%)	10 (35%)	10 (33%)	.707
Part time	17 (29%)	7 (24%)	10 (33%)	
Do not work	22 (37%)	12 (41%)	10 (33%)	
**Monthly household income (Yuan)**				
<1000	22 (37%)	15 (52%)	7 (23%)	.125
1000–1999	16 (27%)	5 (17%)	11 (37%)	
2000–2999	15 (25%)	6 (21%)	9 (30%)	
≥3000	6 (10%)	3 (10%)	3 (10%)	
**HIV infection**				
Years of HIV infection (SD)	2.66 (2.11)	2.03 (1.92)	3.26 (2.14)	**.025**
Receive ART	30(51%)	15(52%)	15(50%)	.895
Duration on ART in months (SD)^a^	32.47(23.26)	23.33(21.49)	41.60(21.91)	**.029**
HIV infection among other family members	17 (29%)	9 (31%)	8 (27%)	.779
HIV infection of partner (yes or unsure)	29 (64%)	17 (81%)	12 (50%)	.060
**Endocrine measures**				
Cortisol level	15.75(7.97)	18.02(9.37)	13.55(5.67)	**.030**
DHEA	36.38(22.52)	33.71(23.26)	38.97(21.87)	.375
DHEA/cortisol	2.59(1.72)	2.08(1.68)	3.08(1.63)	**.024**
**Psychological measures**				
Stressful life events (SD)	20.03(3.28)	21.24 (3.94)	18.87 (1.91)	**.004**
Perceived stress (SD)	32.76(11.12)	37.28 (9.19)	28.40(11.21)	**.002**
Anxiety (SD)	30.31(10.80)	35.31 (12.54)	25.47 (5.67)	**.000**
Depression (SD)	17.04(11.71)	23.69(13.09)	10.60(4.67)	**.000**

^a^Data were available only from the patients who had received ART.

Most background characteristics were similar by stress group with only a few exceptions. High stress group had lower BMI (20.10 vs. 21.82, p < .05), more young children (1.62 vs. .95, p < .05), and shorter duration of HIV infection (2.03 vs. 3.26, p < .05) than low stress group.

### Endocrine characteristics by stress group

**Fig 1:**
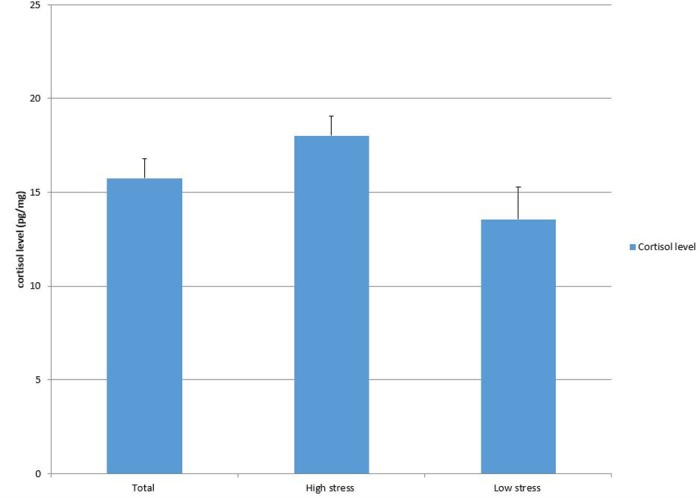
Cortisol level by group.

**Fig 2:**
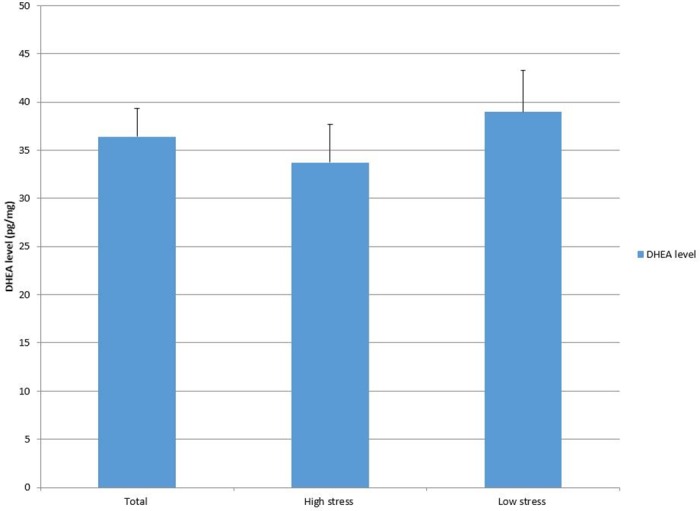
DHEA level by group.

**Fig 3:**
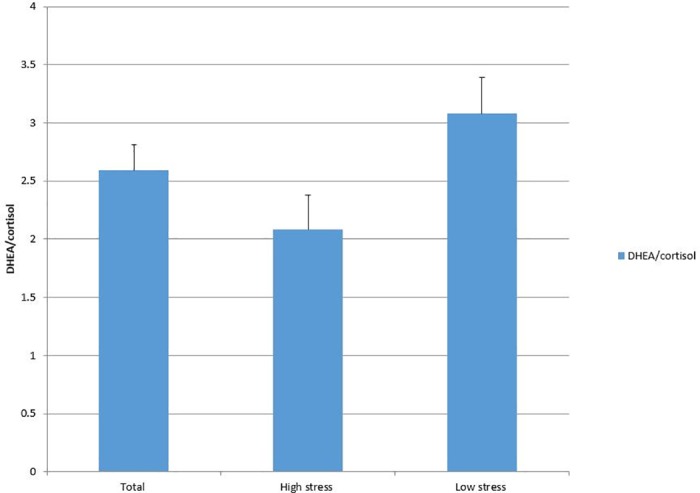
DHEA/cortisol group.

Endocrine measures for the total sample (n = 59) and each stress group are displayed in Figs 1–3. The average hair cortisol level was 15.75 pg/mg (SD = 7.97; RANGE: 4.95, 47.11) among the sample, with significantly higher levels in the high stress group compared to the low stress group (18.02 vs. 13.55, *p* = .03). The mean DHEA among participants was 36.38 with a large variation (SD = 22.52; RANGE: 2.85, 109.37). No significant difference was detected in DHEA between stress groups. The DHEA to cortisol ratio was 2.59 (SD = 1.72; RANGE: .32, 8.59) on average. This ratio was significantly lower in high stress group than in low stress group (2.08 vs. 3.08, *p* = .02).

### Psychological measures by stress group

The means of psychological measures by stress group are presented in Table 1. Compared to the low stress group, the high stress group reported significantly higher chronic stress levels in terms of stressful life events (21.24 vs. 19.87, *p* = .004), perceived stress level (37.28 vs. 28.40, *p* = .002), and anxiety (35.31 vs. 25.47, *p* < .001). In addition, the higher stress group reported significantly higher levels of depression than the low stress group (23.69 vs. 10.60, *p* < .001). This finding confirmed the validity of these self-report measures of psychological stress were as valid among PLWH in China.

### The associations between psychological measures and endocrine measures

According to Pearson correlation statistics, hair cortisol was positively related to anxiety (ρ = .279, p = .033) and perceived stress (ρ = .246, p = .06); DHEA was negatively related to stressful life events (ρ = -.228, p = .082); the DHEA to cortisol ratio was negatively related to stressful life events (ρ = -.395, p = .002), anxiety (ρ = -.221, p = .093) and perceived stress (ρ = -.226, p = .086). Means for each psychological measure by endocrine groups are illustrated in Table 2. Hair cortisol level was significantly associated with anxiety (t-statistic = 2.210, p = .033). It was also positively related to perceived stress although the magnitude did not reach statistical significance (t-statistic = 1.870, p = .067). DHEA was negatively associated with stressful life events (t-statistic = -2.324, p = .025). And the DHEA to cortisol ratio was negatively associated with both stressful life events (t-statistic = -2.888, p = .006) and perceived stress (t-statistic = -2.200, p = .032).

**Table 2:** Psychological measures by level of endocrine measures.

Mean(SD)	Cortisol			DHEA			DHEA/Cortisol		
	Low	High	p-value	Low	High	p-value	Low	High	p-value
**Stressful life events**	19.45(3.70)	20.60(2.76)	.180	20.97(3.91)	19.07(2.14)	**.025**	21.17(3.83)	18.86(2.07)	**.006**
**Perceived stress**	30.07(10.55)	35.37(11.21)	.067	34.17(10.85)	31.31(11.40)	.328	35.80(10.35)	29.62(11.18)	**.032**
**Anxiety**	27.28(6.27)	33.23(13.31)	**.033**	30.67(9.73)	29.93(11.97)	.796	32.27(10.31)	28.28(11.10)	.158
**Depression**	14.59(9.64)	19.40(13.15)	.115	16.17(10.51)	17.93(12.96)	.567	17.63(11.11)	16.41(12.47)	.693

### The effect of ART on endocrine measures

Gender, religion affiliation, hair treatment in past month were not significantly associated with the endocrine measures based on t-tests; and there was no significant association between endocrine measures with BMI, age, education attainment, number of children or duration of HIV infection. Therefore, these potential covariates were not included in the GLM analysis. Table 3 shows the results of GLM analysis in which the effects of ART status and its interaction with stress group were assessed simultaneously. The multivariate test in the GLM analysis suggested a significant main effect for stress group (F = 2.903, p = .043) but not for ART status. The interaction term between stress group and ART status was significant at neither multivariate level nor univariate level. Univariate tests showed that stress group was significantly associated with hair cortisol (F = 4.859, p = .032) and DHEA to cortisol ratio (F = 5.173, p = .027). However, stress group was not significantly related to DHEA. ART was not significantly associated with any of the endocrine measures in univariate tests.

**Table 3:** GLM results.

F-statistics	Main effect		Interaction
	Stress group (S)	ART Status (A)	S*A
**Multivariate test**	2.903(p = .**043**)	1.109(p = .354)	<1
**Univariate test**			
Cortisol	4.859(p = .**032**)	2.740(p = .104)	<1
DHEA	<1	<1	<1
DHEA/cortisol	5.173(p = .**027**)	1.439(p = .235)	<1

## Discussion

Employing a known-groups validation methodology, our data confirmed the endocrine measures as valid biomarkers of chronic stress among PLWH in China. Individuals with higher level of psychological stress displayed higher levels of hair cortisol and a lower DHEA to cortisol ratio than individuals with lower level of psychological stress. This result is consistent with existing literature among other populations. One recent systematic literature review on hair cortisol and stress exposure indicated that effect sizes for the connections between hair cortisol and chronic stress were mostly medium and large [38]. Previous studies also have shown that the ratio of DHEA to cortisol is related to chronic stress, which was either measured by psychological scales including Perceived Stress Scale (PSS)[39], Hamilton Depression Rating Scale (HAM-D)[40], or determined by clinical diagnosis [41]. One study among HIV-infected men reported that the DHEA to cortisol ratio was dramatically altered in the HIV-infected men, particularly under conditions of malnutrition and lipodystrophy [27]. Some studies have also demonstrated that DHEA in serum and saliva are related to long-term psychological stress [19, 42]. However, no significant difference in DHEA level was detected between stress groups in the present study. Future studies are needed to explore the relationship between DHEA level in hair and chronic stress among PLWH.

We also found that the endocrine measures were associated with self-reported psychological measures. For instance, hair cortisol level was significantly related to anxiety and DHEA to cortisol ratio was significantly associated with stressful life events and perceived stress. Existing studies have reported mixed findings regarding the relationship between hair cortisol and mental health [38]. Some studies indicate that hair cortisol increased with higher level of stressful life events, depression, and posttraumatic stress disorder [43–45]. However, a recent literature review suggested that the effect sizes for associations between hair cortisol and mental health were small to medium [38]. The results in the current study provide additional empirical evidence for the connections between endocrine function and mental health. While DHEA may be related to anxiety [46] and depression [39, 41], DHEA was negatively related to stressful life events but not to other psychological measures in the present study. The relationship between DHEA and mental health among PLWH in China needs to be confirmed in the future by empirical studies with a larger sample.

Limited empirical studies have examined the effect of ART status on hair concentrations of steroid hormones among PLWH. Our study provided new preliminary data in this regard by demonstrating that the associations between the endocrine measures and psychological stress among PLWH did not differ by ART uptake status. Research on how HIV infection may affect HPA axis activity suggests that many of the anti-retroviral and other medications for opportunistic infection can contribute to HPA axis dysfunction [47]. For example, it is not uncommon for HIV patients on ART to report altered adrenocortical function and adrenal insufficiency [48, 49]. However, it was reported that the change of serum cortisol and DHEA level was not influenced by ART treatment [27]. More empirical and etiological studies on the potential moderation effect of ART will shed light on the stress-related psychopathology and pharmacology among PLWH.

The findings of this study should be interpreted with caution given a number of potential limitations. First, our analysis was based on a purposely recruited small sample without a healthy control group. The small sample size of this exploratory study limits the statistic power of the analysis. The sampling approach may also impede the representativeness of the sample and limit our ability to generalize results to other PLWH populations. Second, the assignment of participants into different stress group was not based on clinical diagnosis but psychological interview. Although the assessment interview was done by a licensed psychological counselor with extensive working experience in HIV case management and care, the assessment and assignment may be subject to some subjective bias. However, the accuracy of such assignment was partly supported by the significant differences in the self-reported stress measures between the two groups. Third, we employed endocrine measures in human hair to assess the chronic stress level in the past one month in addition to utilizing several psychological scales (e.g., depression and anxiety) that used the past one week as an assessment time-frame. The discrepancy of the time-frame of the assessment tools may contribute to weak correlations between the endocrine measures and some of the self-reported psychological measures [38]. Fourth, although we tried to comprehensively assess and control the potential confounders in the current study, data were not available on other variables that may also potentially influence endocrine release, such as AIDS disease stages among PLWH. The findings should be confirmed by future study that can control for these variables.

Despite these potential limitations, the current study presents the first effort to investigate the relationship between hair concentrations of endocrine measures, chronic stress, and mental health among PLWH in China. Based on the results, endocrine measures in hair can be used as promising biomarkers to significantly enhance current understanding of the role of steroid hormones in psychoimmunological study. Valid biomarkers of chronic stress among PLWH yield important implications for clinical practice and psychological interventions. Hair concentrations of cortisol and DHEA to cortisol ratio may provide a means to track the effect of psychological processes (including chronic stress) on the long-term HPA axis activity. It constitutes a unique retrospective approach to assess baseline endocrine level before the onset of a major life event or psychological problem (e.g., anxiety or depression). Hair cortisol and DHEA to cortisol ratio thus could be reliable biomarkers incorporated in stressor identification, early diagnosis of mental health problems, and monitoring of psychological wellbeing in clinical settings of HIV counseling and care. They could also be objective indicators to monitor and evaluate the efficacy of psychological interventions or psychotherapeutic treatment.

Hair analysis has been recently introduced and applied in HIV prevention and treatment research. For example, antiretroviral medicine concentrations in hair have been used in assessment of long-term ART medication adherence [50–53]. To extend the utility of endocrine measures in psychological research among PLWH, future studies are needed to focus on several aspects. First, more etiological research is needed on the mechanisms of how chronic stress may affect hair cortisol and DHEA level among PLWH. For example, future studies need to explore what initiates cortisol or DHEA release, to what extent hair cortisol or DHEA can be changed, and whether and how the disease course of HIV/AIDS and ART treatment influence the two endocrine hormone levels.

Second, given the influence of HIV infection and AIDS disease on HPA axis activity, the normal ranges of hair cortisol or DHEA among PLWH may vary from that of healthy adults. It is necessary to form reference values or norm values of these biomarkers for the comparability between different sub-groups of PLWH. More empirical evidence on hair cortisol and DHEA among PLWH are needed, and it is also critical to assimilate the laboratory techniques and establish a “gold standard” of the extraction and analysis [38].

Third, longitudinal studies with large samples are needed to establish the linkages between different psychological problems and long-term changes in patterns of hair cortisol and DHEA. Existing literature indicates that the change patterns of hair cortisol levels may vary in accordance with different phases of psychological disorders. For instance, Luo and colleagues noted increased hair cortisol concentrations after one month and decreased cortisol level after seven months following an earthquake among adolescents with PTSD [54]. Longitudinal studies will contribute to development of a comprehensive picture of hair concentrations of endocrine hormones through tracking the psychopathological processes that underline these variations.

Last but not least, the cognitive processes in stress response and its potential association with hair cortisol and DHEA level should not be ignored in further studies. The cognitive appraisal of a stressor partly consists of the stress response itself [55]. Analysis of hair cortisol and DHEA may provide options to identify protective factors at individual level (resilience, coping) and interpersonal level (social relationship and social support), as well as to explore the psychological effects of these factors on the physiological stress response. Future studies should examine whether personality aspects and social-emotional functioning of PLWH may affect their stress responses as measured by hair cortisol and DHEA.

In summary, our study confirms that hair cortisol and DHEA to cortisol ratio can be valid biomarkers of chronic stress among PLWH and demonstrates their associations with several self-reported psychological measures. Hair sample collection is noninvasive and does not require specific skills, sterile equipment, or specialized storage conditions [56]. Hair samples can be stored for long periods prior to analysis at room temperature and delivered without any biohazardous precautions or cold chain requirements [52]. These features may make hair cortisol and DHEA feasible biomarkers in resource-constrained settings, where hair collection has proved highly acceptable [57, 58]. Therefore, hair cortisol and DHEA analysis hold great promise to accurately assess long-term levels of chronic stress, which could greatly contribute to early diagnosis and intervention of mental health diseases and the improvement of psychosocial wellbeing among HIV-infected people.

## Supporting Information

S1 Dataset SPSS data set for this paper. (SAV)
